# Probiotics in Inflammatory Bowel Disease: Are We Back to Square One?

**DOI:** 10.7759/cureus.10247

**Published:** 2020-09-04

**Authors:** Suvarna Rekha Puvvada, Enkhmaa Luvsannyam, Dhara Patel, Zaira Hassan, Pousette Hamid

**Affiliations:** 1 Internal Medicine, California Instititute of Behavioral Neurosciences & Psychology, Fairfield, USA; 2 Plastic Surgery, California Instititute of Behavioral Neurosciences & Psychology, Fairfield, USA; 3 Family Medicine, California Instititute of Behavioral Neurosciences & Psychology, Fairfield, USA; 4 Neurology, California Instititute of Behavioral Neurosciences & Psychology, Fairfield, USA

**Keywords:** ibd, probiotics, microbiome

## Abstract

Inflammatory bowel disease (IBD) is a chronic inflammatory condition of the alimentary tract whose incidence has been increasing over the past few years. Even though there is a complex interplay of several factors in the pathogenesis of IBD, a decrease in the diversity of intestinal microbiome is commonly found in patients. Extensive research is directed towards the alteration of this microbiome to improve the symptoms of IBD. Probiotics, prebiotics, antibiotics, and diet are studied in this regard extensively. Among them, probiotics have gained more interest as some of the studies showed them to be effective in decreasing gut inflammation in vitro and in vivo. Although there is no cure for IBD as of today, the available medications do decrease gut inflammation and help prolong remission and decrease relapse rates. But their side effects preclude their long-term use. Probiotics may be a ray of hope among IBD patients as they are apparently safe. This article reviews each of the available literature from the past 10 years to see if there is any certain role of probiotics in induction, maintenance of remission, prevention of relapse, and improvement in the quality of life in IBD patients.

## Introduction and background

Inflammatory bowel disease (IBD), which consists of two patterns, ulcerative colitis (UC) and Crohn’s disease (CD), is a chronic inflammatory condition. The etiology of IBD is multifactorial. Genetics, immunological and environmental factors along with disturbances in the intestinal barrier are implicated in the pathogenesis of IBD [1]. Out of these, environmental factors like smoking, diet, microbiota, and medications are modifiable and have been extensively studied in recent times [1]. The reason for this growing interest is, despite the advances in genetics, only about 25% of IBD cases are known to be heritable. The epidemiological trends in developing countries after industrialization suggest that environmental factors may play a pivotal role in causing intestinal inflammation in genetically susceptible individuals [2,3]. Second, dysbiosis is documented to play a major role in IBD pathogenesis [4]. Ever since studies on dysbiosis as a cause for IBD started blooming, researchers parallelly have been studying the factors that could modify the microbiome. Third, though the available drug therapies, which work by blocking the immune system, are shown to be effective in remission of IBD, there has been a concern regarding their side effects [5]. Also, gut microbiota, along with taking part in pathogenesis, influence the efficacy of certain drug therapies, such as anti-tumor necrosis factor-α (anti-TNF-α), steroids, etc. [6,7]. So there is a constant search for therapies that would induce and continue remission in IBD patients with minimum side effects. In this regard, probiotics have gained interest in recent times.

Probiotics by definition are living microorganisms that exert beneficial effects on the host by modifying the intestinal microbiota [8]. Since the first-ever concept of probiotic in the 20th century as speculated by Metchnikoff, that life could be prolonged by manipulating the intestinal microbiome with friendly bacteria *Lactobacillus bulgaricus* found in yogurt, a lot of light has been shed on the mechanism of action of probiotics [9]. It has been proved that once ingested, probiotics work by decreasing harmful microbiota, increasing protective microbiota, improving gut epithelial barrier function by producing various metabolites like short-chain fatty acids, and modulating the immune system [10].

There is always a debate regarding the benefit of probiotic usage in IBD patients. A meta-analysis conducted by Ganji‐Arjenaki and Rafieian‐Kopaei showed the efficacy of probiotics in UC, but not in CD [11]. Another meta-analysis done by Derwa et al. concluded that probiotics are as effective as 5-aminosalicylic acid (5-ASA) in preventing relapse in inactive UC and the probiotic VSL#3 is especially effective in inducing remission in UC patients [12]. A study conducted by Cordina et al. showed that probiotics are popularly being prescribed by physicians in patients with gastrointestinal disorders, especially in IBD [13]. Many commercial over-the-counter food products that claim to contain probiotics are extremely popular among the public. In this regard, we are trying to identify and review the available data from the past 10 years and give a bird’s eye view on the usage of probiotics in IBD. By this review, we aim to answer a few questions such as whether probiotics are effective in achieving remission, preventing relapse, and decreasing symptoms/inflammation in IBD. If so, whether they are equally useful in various types of IBD, and also, the desired direction of future studies.

## Review

We conducted a search of the literature in PubMed, Google Scholar, and Medical Subject Headings (MeSH) on Demand using search terms such as probiotics, ulcerative colitis, Crohn’s disease, and inflammatory bowel disease. The studies included are randomized control trials including adults (age >18 years) with active or quiescent IBD, using either single- or multi-strain probiotics compared to placebo, assessing the outcome measure of remission, relapse, or quality of life (QOL) in the past 10 years. Animal studies, studies with population aged <18 years, and studies not measuring the above-mentioned outcomes were excluded from this review.

Our search got us 13 relevant studies that we reviewed in this article. The included studies (n=13) are summarised in Table 1.

**Table 1:** Summary of included studies (n=13) UC: ulcerative colitis; CD: Crohn's disease; CFU: colony-forming unit; CRP: C-reactive protein; ESR: erythrocyte sedimentation rate; Hb: hemoglobin; IBD: inflammatory bowel disease; QOL: quality of life; FCAL: fecal calprotectin; UCDAI: Ulcerative Colitis Disease Activity Index; EcN: Escherichia coli Nissle; CDAI: Crohn's Disease Activity Index; DC: dendritic cell; TLR: Toll-like receptor; IL: interleukin; ITT: intention to treat; DAI: disease activity index; EI: endoscopic index; CFU: colony-forming unit

Author and year	Location	Disease	Pattern	Probiotic used	Dose of probiotic	Intervention	Duration of probiotic given	Results
Yilmaz et al., 2019 [14]	Turkey	UC and CD	Active	Kefir (1 ml contains 2 x 10^10 ^CFU Lactobacillus)	400 ml twice a day	Probiotic vs respective disease controls	4 weeks	Statistically significant increases in the Lactobacillus load in both UC and CD patients; a decrease in CRP, ESR, bloating scores and an increase in Hb, feeling good scores in the probiotic arm
Bjarnason et al., 2019 [15]	United Kingdom	UC and CD	Quiescent or mild active	Symprove™ (Lactobacillus plantarum NCIMB 30173, Lactobacillus rhamnosus NCIMB 30174, Lactobacillus acidophilus NCIMB 30175 and Enterococcus faecium NCIMB 30176)	1 ml/kg/day	Probiotic vs placebo	4 weeks	No difference in IBD QOL in both arms. FCAL levels decreased in UC but not in CD compared to placebo.
Matsuoka et al., 2018 [16]	Japan	UC	Inactive	Lactobacillus acidophilus (1 billion bacteria) and Bifidobacterium breve strain Yakult (10 billion bacteria)	100 ml/day	Probiotic vs placebo	48 weeks	There was no significant difference in relapse-free intervals nor the incidence of relapse in both arms.
Vejdani et al., 2017 [17]	Iran	UC	Active Mild to moderate	Lactobacillus casei ATCC PTA-3945 capsule (5 x 10^5 ^organisms)	One capsule twice a day	Probiotic vs placebo	8 weeks	No significant difference in remission and relapse rates between two arms
Tamaki et al., 2016 [18]	Japan	UC	Active	Bifidobacterium longum 536 (BB536 2-3 x 10^11 ^organisms)	Three times a day	Probiotic vs placebo	8 weeks	No statistically significant change in remission rates in two arms; significant decrease in UCDAI scores, Rachmilewitz EI, and Mayo sub-score in the probiotic arm
Yoshimatsu et al., 2015 [19]	Japan	UC	Inactive	Bio-Three tablet (10 mg of Clostridium butyricum TO-A, 2 mg of Streptococcus faecalis T-110, and 10 mg of Bacillus mesentericus TO-A)	Three tablets three times a day	Probiotic vs placebo	12 months	Significantly fewer relapse rates in the probiotic arm at 3 and 6 months; no significant difference in remission rates at 12 months in both arms
Fedorak et al., 2015 [20]	Canada	CD	Post-surgery	VSL#3 sachet (four strains of Lactobacillus, three strains of Bifidobacterium, and one strain of Streptococcus salivarius subspecies thermophilus)	One sachet twice a day	Probiotic vs placebo	365 days	No statistically significant difference in recurrence rates and severity proportion at 90 days in two arms and also aggregate severe recurrence rates at 365 days; statistically significant decrease in mucosal inflammatory cytokine levels in the VSL#3 arm at 90 days
Petersen et al., 2014 [21]	Denmark	UC	Active	EcN	100 mg once a day for four days followed by 100 mg twice a day	Ciprofloxacin/ placebo for one week followed by EcN/placebo for seven weeks	7 weeks	Statistically significant fewer remission rates and lack of mucosal healing in placebo followed by EcN compared to other groups. Groups receiving EcN reached remission less frequently than groups not receiving EcN
Bourreille et al., 2013 [22]	France	Crohn’s disease	Inactive	Saccharomyces boulardii	1 g/day	Probiotic vs placebo	52 weeks	No statistically significant difference in mean CDAI scores, ESR, or CRP at 52 weeks. No beneficial effects of the probiotic
Wildt et al., 2011 [23]	Italy	UC	Inactive	Probio-Tec AB-25 capsule (1.25 x 10^10^ CFU of Bifidobacterium animalis strain BB-12 and 1.25 x 10^10^ CFU of Lactobacillus acidophilus strain La-5)	Two capsules three times a day	Probiotic vs placebo	52 weeks	No statistically significant difference in remission after one year and relapse rates in two arms
Tursi et al., 2010 [24]	Italy	UC	Active	VSL#3	Two sachets twice a day	Probiotic vs placebo	8 weeks	Statistically significant improvement of at least 50% in UCDAI scores and a decrease in UCDAI scores from baseline in the probiotic arm; no statistically significant difference in the induction of remission in two arms
Ng et al., 2010 [25]	United Kingdom	UC	Active	VSL#3	Two sachets twice a day	Probiotic vs placebo	8 weeks	Statistically significant decrease in intestinal DC TLR-2 expression, IL-12p40 production and an increase in IL-10 production
Matthes et al., 2010 [26]	Germany	UC	Active	EcN 1917 (10^8 ^organisms/ml)	10, 20, 40 ml enemas	Probiotic vs placebo	12 weeks	No statistically significant difference in the number of responders (DAI<2) in both arms after ITT analysis. Statistically significant dose-dependent efficacy was noted in per-protocol response rates.

As IBD has no effective cure as of today, patients often resort to all available alternative treatments for this chronic illness. Probiotics happen to be the most commonly used alternative products for IBD treatment. Studies show that an estimated 3.9 million American adults used probiotics and prebiotics in the year 2015, which is four times that used in the year 2007 [27,28]. There is no strict regulation on the marketing of these over-the-counter products as they are considered as food products but not as medication by US FDA [29].

Efficacy of probiotics in induction, maintenance of remission, and prevention of relapse in UC

Traditionally, anti-inflammatory medications have been used for the induction and maintenance of remission in UC patients. The 5-ASA class of medications has been used as the first-line medication for the induction of remission in mild to moderate cases. Since the evolution of the concept of probiotics, they have been studied as a safe alternative. Ng et al. conducted a study with 28 active UC patients who were randomized to either VSL#3 or placebo groups for eight weeks. The results showed that 10 of 14 and 5 of 14 achieved clinical response in probiotic and placebo groups, respectively, but the difference was not statistically significant [25]. Matthes et al. randomized 90 UC patients with the active disease into *Escherichia coli *Nissle (EcN) and placebo groups. Treatments were given through enemas for two weeks and the patients were followed for eight weeks. EcN was given in doses of 10, 20, and 40 ml. They did not find any statistically significant difference in the number of responders (disease activity index<2) in both arms after intention-to-treat (ITT) analysis (p=0.4430, two-sided). Statistically significant dose-dependent efficacy was noted in per-protocol respond rates: EcN 40 ml, 52.9%; 20 ml, 44.4%; 10 ml, 27.3%; placebo, 18.2% (p=0.0446, two-sided) [26]. Tamaki et al. conducted a study on mild to moderate UC patients and found that there was a significant decrease in Ulcerative Colitis Disease Activity Index (UCDAI) scores in the *Bifidobacterium longum *(BB536) group from baseline after eight weeks (p<0.01), but this was not seen in the placebo group (p=0.88). Also, the Rachmilewitz endoscopic score and Mayo sub-score were significantly decreased in the probiotic arm but not in the placebo arm. However, the clinical remission defined as UCDAI <2 at eight weeks did not differ in the probiotic versus placebo group (63% vs 52%, p=0.395) [18]. Vejdani et al. conducted a study on mild to moderate UC patients by randomizing them to *Lactobacillus casei* or placebo group while continuing the conventional therapy in both the groups. They found that there is no significant difference between probiotic versus placebo group in remission rates (82% vs 76% at ITT analysis, p=1.00, and 100% vs 81.2% at per-protocol analysis, p=0.23) and relapse rates (14.3% vs 26.7% at ITT, p=0.65, and 16.7% vs 33.3% at per-protocol analysis, p=0.64). The same was the case with the mean time to remission and relapse between the two groups (p=0.11 and p=0.51) [17]. Wildt et al. conducted a study in UC patients in remission using Probio-Tec AB-25 and concluded that there was no significant difference in the percentage of patients maintaining remission after one year in the probiotic versus placebo group (25% vs 8%, p=0.37) and also in median time to relapse (125.5 days vs 104 days, p=0.683) [23]. Matsuoka et al. randomized 195 patients with quiescent UC into BFM fermented milk or placebo groups for 48 weeks. They did an interim analysis and the results showed no significant difference in the incidence of relapse or relapse-free survival (p=0.643, a hazard ratio of 1.16% with 95% CI 0.63-2.14, log-rank test) or maintenance of remission between two arms. They couldn’t find any effect of probiotics on intestinal microbiota as evident from fecal samples [16]. Tursi et al. conducted a study on 65 active UC patients randomizing them to VSL#3 or probiotic. The UDAI scores greater than or equal to 50% were more in the probiotic compared to the placebo group (63.1 vs 40.8; per-protocol p=0.010, 95% CI 0.51-0.74; ITT). A significant improvement of the UDAI score greater than or equal to 3 was seen in the VSL#3 group (p=0.017). At the same time, no difference was seen in the rate of disease activity and endoscopic scores in both groups. Though the remission rates were higher in the VSL#3 group, it was not statistically significant [24].

In contrast to the above-mentioned studies, a study done by Yoshitmasu et al. in 60 UC patients in remission using Bio-Three and placebo for 12 months showed that by the end of three months, the relapse rates in Bio-Three were less compared to placebo (0.0% vs 17.4%,p=0.036), but there was no difference at six and nine months. There was no difference in remission rates at 12 months between Bio-Three (69.5%) and placebo (56.6%) groups, p=0.248 [19]. Another study was done by Peterson et al. in patients with active UC, where patients were initially randomized into ciprofloxacin or placebo group for one week followed by EcN or placebo for seven weeks. They found that patients who received placebo/EcN had lesser remission compared to the placebo/placebo group (54% vs 89%, p<0.05). Also, the placebo/EcN group had significantly more number of withdrawals, 11/25 (44%) compared to other groups 15/75 (20%), p<0.05. Mucosal healing in placebo/EcN (29%) was less compared to other groups (p<0.05) [21].

We found that most of the studies showed that probiotics have no role in achieving remission in UC patients. Although there was a decrease in disease activity index scores compared to baseline in a few studies, there appears to be no significant decrease to meet the criteria to define remission. The relapse rates and relapse-free intervals are also not affected by probiotics.

Efficacy of probiotics in relapse in CD

There is very little data available on the role of probiotics in the relapse of CD. The clinical and endoscopic relapse rates for low versus high-risk CD 18 months after surgery are 20 and 30 versus 50 and 80, respectively [30]. Fedorak et al. conducted a study in CD patients within 30 days of ileocolic resection and anastomosis assigning them to the VSL#3 or placebo group. Endoscopy was done at 90 days and they found that there is no difference between VSL#3 and placebo groups in terms of percentage of patients with severe endoscopic lesions (9.3% vs 15.7%, p=0.19). The patients with no or mild endoscopic lesions at 90 days from both the groups who were continued with probiotics till 365 days and who had severe endoscopic lesions at 365 days showed no difference between early versus late starters of probiotics (10% vs 26.7 %, p=0.09). Also, there was no significant difference in aggregate severe recurrence rates on 90 and 365 days as evidenced by 20.5% and 42.1% of subjects in the early versus late VSL#3 group, respectively [20]. Bourreille et al. conducted a study on 165 CD patients who were on remission and randomized them to either *Saccharomyces boulardii* or placebo group for 52 weeks. They found that there was no significant difference between the probiotic versus placebo group in the percentage of relapse (47.5% vs 53.2%), the median time to relapse (40.7 vs 39 weeks), CD activity index scores, erythrocyte sedimentation rate, and median C-reactive protein (CRP) levels [22].

As only two out of 13 studies were done on the efficacy of probiotics in relapse of CD, it is to be noted that there is not much evidence to support any role of probiotics in decreasing relapses in CD.

Efficacy of probiotics in QOL in IBD patients

Improvement in QOL is an important aspect of the treatment of IBD patients. Patients with active disease have a poor QOL regardless of the disease subtype. Yilmaz et al. conducted a study using probiotic kefir on 45 active IBD patients (25 in the treatment group and 20 in the control group), out of which 25 were UC and 22 CD. They observed a significant increase in the *Lactobacillus* load in feces of both UC (p=0.001) and CD (p=0.005) patients by four weeks. In patients with CD, there was a significant reduction in bloating (p=0.012) and improvement in feeling good scores (p=0.032) in the last two weeks, a decrease in CRP levels (p=0.015), and an increase in hemoglobin levels (p=0.024) at four weeks in the kefir group. They also found that the rate of feeling good score in CD was significantly more compared to UC (p=0.019) [14]. In a study by Bjarnason et al., 81 UC and 61 CD patients were randomized to receive either Symprove™ (Symprove Ltd, Farnham, UK) or placebo for four weeks. They observed no significant change in IBD QOL and clinical disease activity before and after treatment between the probiotic and placebo groups. A post hoc analysis of fecal calprotectin levels in the probiotic group showed a significant decrease (p=0.011, for t-test, and p=0.001, for Wilcoxon’s) after four weeks in UC but not CD patients. There were 0/40 relapses in the probiotic arm and 4/41 relapses in the placebo arm in UC patients. There were no relapses seen in CD patients in both arms [15]. Fedorak et al. found that the patients on VSL#3 had decreased mucosal inflammatory cytokines at day 90 compared to the placebo group (p<0.05). They also noticed that the CD activity index and IBD QOL were similar in VSL#3 and placebo groups [20]. Ng et al. found significant decreases in dendritic cell Toll-like receptor-2 expression (p<0.05) and proinflammatory cytokine interleukin-12p40 production (p<0.05) in the VSL#3 arm, which was not seen in placebo [25].

Two out of three studies showed improvement and one showed no change in QOL in IBD patients. Based on these findings, we can say that probiotics might have a positive effect on QOL in patients with IBD.

Limitations

Each of the studies was conducted on small populations over a short duration of time. These factors ultimately affect the power of the study. Also, there is no consistency in strains, their doses, and route of administration in these studies that precludes us from combining these results. We included studies from the past 10 years and could have missed data from previous years.

Future directions

Future randomized control trials should aim to design a protocol to test the efficacy of individual probiotic strains by conducting larger trials in different ethnic groups to identify the doses that will modify the microbiome and then combine the results to formulate a guideline for their use in IBD. This requires an immense amount of testing, time, funding, and regulation on the usage of probiotics. We hope to find answers for a few questions in the future, such as what is the bioavailability of the probiotics? Which route of administration is better? What is the exact pathogenesis of IBD that causes a change in the microbiome? How does a combination of different strains affect the efficacy of individual strain?

## Conclusions

The relationship between the intestinal microbiome and its host is complex. Probiotics have the ability to manipulate the environment of the intestinal microbiome by their competitive nature to eliminate pathogens. Previous studies show that they have anti-inflammatory and immunomodulatory effects, but their results in IBD remission and relapse are heterogeneous and inconsistent. Although probiotics are considered a safe complementary treatment for IBD, their effect on induction, maintaining remission and relapse is questionable even today as there is no strong evidence supporting their use. Therefore, this leaves us with the fact that the same questions are staring at us for definitive answers after all these years.
